# Putting a bit into the polo-box domain of polo-like kinase 1

**DOI:** 10.1186/s40543-015-0069-y

**Published:** 2015-10-14

**Authors:** Jung-Eun Park, Tae-Sung Kim, Lingjun Meng, Jeong K. Bang, Bo Y. Kim, Kyung S. Lee

**Affiliations:** Laboratory of Metabolism, Center for Cancer Research, National Cancer Institute, National Institutes of Health, 9000 Rockville Pike, Building 37, Room 3118, Bethesda, MD 20892 USA; Division of Magnetic Resonance, Korea Basic Science Institute, 804-1, Yangcheong Ri, Ochang, 363-883 Chungbuk Republic of Korea; World Class Institute, Korea Research Institute of Bioscience and Biotechnology, Ochang, 363-883 Republic of Korea

**Keywords:** Polo-like kinase 1, Polo-box domain, Inhibitor, Cancer therapy

## Abstract

Polo-like kinase 1 (Plk1) plays key roles in regulating various mitotic processes that are critical for cellular proliferation. A growing body of evidence suggests that Plk1 overexpression is tightly associated with the development of human cancers. Interestingly, various types of cancer cells are shown to be addicted to a high level of Plk1, and the reversal of Plk1 addiction appears to be an effective strategy for selectively killing cancer cells, but not normal cells. Therefore, Plk1 is considered an attractive anticancer drug target. Over the years, a large number of inhibitors that target the catalytic activity of Plk1 have been developed. However, these inhibitors exhibit significant levels of cross-reactivity with related kinases, including Plk2 and Plk3. Consequently, as an alternative approach for developing anti-Plk1 therapeutics, substantial effort is under way to develop inhibitors that target the C-terminal protein–protein interaction domain of Plk1, called the polo-box domain (PBD). In this communication, I will discuss the pros and cons of targeting the PBD in comparison to those of targeting the ATP-binding site within the kinase domain.

## Review

Protein phosphorylation by protein kinases represents a fundamental mechanism underlying diverse biochemical and cellular processes that are important for the proliferation of eukaryotic cells (Hanks et al. 1988). Protein kinases are a family of enzymes that catalyze the transfer of the gamma phosphate from adenosine triphosphate (ATP) to a protein substrate and, as a result, induce a change in the conformation and function of the protein substrate. A large body of evidence suggests that deregulating this process can lead to various pathological disorders in humans, including cancers (Lahiry et al. 2010). Therefore, deregulated protein kinases may represent attractive targets for the development of therapeutics against various human disorders. However, unlike initial expectations, targeting protein kinases has proven difficult largely because of the similarities in their primary sequences and conserved structural motifs around the ATP-binding site. Nevertheless, recent advances in our understanding of this family of enzymes have allowed us to overcome these obstacles and develop a sizable number of clinically applicable therapeutic agents. According to the Protein Kinase Inhibitors in Oncology Drug Pipeline Update 2015, small-molecule inhibitors were reported against nearly half of a total of 518 cellular protein kinases. Among them, more than 35 inhibitors are approved by the US Food and Drug Administration for clinical applications, and approximately 500 inhibitors are currently in clinical trials for further development.

Although developing inhibitors that target the catalytic activity of a protein kinase has become a prevailing method, various efforts are under way to develop inhibitors that target a functionally critical protein–protein interaction domain of a kinase. This newly emerging strategy, which is thought to yield a higher level of specificity than conventional ATP analog inhibitors, may lead to the development of a different class of inhibitors that could be used either alone or in combination with available catalytic inhibitors to achieve increased drug efficacy.

### Polo-like kinases

**Fig. 1:**
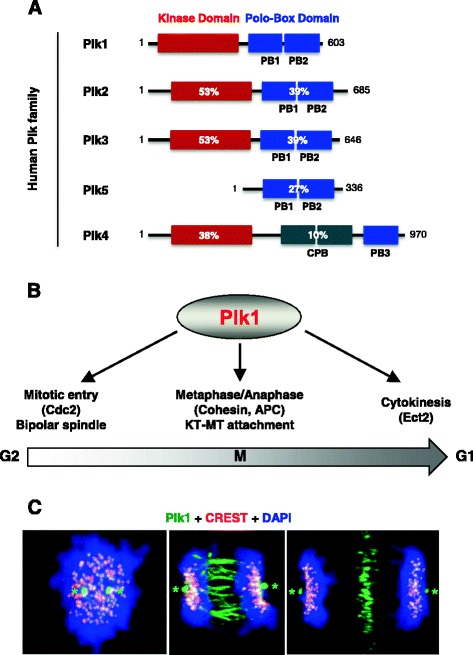
The human Plk family and the function and subcellular localization of Plk1 during the cell cycle. **a** A schematic diagram illustrating the structures of the human Plk family. Sequence identity (%) with Plk1 is shown. *PB1* polo-box motif 1, *PB2* polo-box motif 2, *CPB* cryptic polo box, *PB3* polo-box motif 3. Numbers, amino acid residue numbers for each Plk. **b** A schematic diagram depicting the mitotic functions of Plk1 from G2/M transition to cytokinesis. **c** Subcellular localization of Plk1 in HeLa cells during the cell cycle. Kinetochore-localized Plk1 signals are colocalized with CREST antigens. *Asterisks* centrosomes. These images were originally published in Journal of Biological Chemistry. Seong YS, et al. A spindle checkpoint arrest and a cytokinesis failure by the dominant-negative polo-box domain of Plk1 in U-2 OS cells. 2002; 277(35):32282-93. © the American Society for Biochemistry and Molecular Biology

Polo-like kinases (collectively known as Plks) belong to the evolutionarily conserved polo subfamily of Ser/Thr protein kinases that play pivotal roles in cell proliferation, differentiation, and adaptive responses (see reviews; Winkles and Alberts 2005; Petronczki et al. 2008; Archambault and Glover 2009; Zitouni et al. 2014). In mammalian cells, five Plks (Plk1–5) were identified to date (Fig. 1a), and they exhibit distinct tissue distributions and physiological functions. Except for Plk4, these members contain a signature domain, called the polo-box domain (PBD), which is composed of two motifs with significant homology—polo-box 1 (PB1; residues 405–494 in Plk1) and polo-box 2 (PB2; residues 505–598 in Plk1). Plk4 contains the distantly related cryptic polo box (CPB) and PB3 domains, which interact with its binding targets (Leung et al. 2002; Park et al. 2014).

Among them, Plk1 has drawn a lot of attention because of its tight association with tumorigenesis in human cells. Various studies have shown that Plk1 is highly expressed during the G2 and M phases of the cell cycle (Golsteyn et al. 1995; Lee et al. 1995), and it plays an important role in regulating mitotic entry, centrosome maturation and bipolar spindle assembly, metaphase/anaphase transition, and cytokinesis (Winkles and Alberts 2005; Petronczki et al. 2008; Archambault and Glover 2009; Zitouni et al. 2014) (Fig. 1b). Consistent with the multitude of Plk1 functions, Plk1 has been shown to localize to distinct subcellular structures, such as centrosomes, kinetochores, and midzones/midbodies, in a temporally and spatially regulated manner (Holtrich et al. 1994; Golsteyn et al. 1995; Lee et al. 1995; Arnaud et al. 1998; Seong et al. 2002) (Fig. 1c). The PBD is largely responsible for directing its catalytic activity of Plk1 to specific subcellular locations (Lee et al. 1998; see review; Park et al. 2010) via its capacity to interact with a phosphorylated Ser/Thr motif, thereby bringing the enzyme in close proximity to its binding targets or substrates localized at these sites (Cheng et al. 2003; Elia et al. 2003; Lowery et al. 2004; Park et al. 2010). As expected, the function of Plk1 PBD is essentially required for proper mitotic progression (Lee et al. 1998, 1999; Seong et al. 2002; Hanisch et al. 2006). As of today, a large number of PBD-binding proteins critically required for various Plk1-dependent mitotic events have been isolated and characterized (Park et al. 2010). Thus, the PBD serves as an essential cis-acting element that mediates various Plk1-dependent biochemical steps and cellular processes at specific subcellular structures.

Distinct from the roles of Plk1 during the late stage of the cell cycle, Plk2 appears to be transiently expressed in G1 and contributes to proper S-phase entry (Simmons et al. 1992; Ma et al. 2003a, b). Other studies showed that Plk2 plays a role in maintaining cell viability after spindle poisoning (Burns et al. 2003). Interestingly, Plk3 is expressed throughout the cell cycle (Chase et al. 1998) and has been implicated in responding to DNA damage and cellular stress (Donohue et al. 1995; Xie et al. 2001a, b, 2002, 2005; Bahassi et al. 2002). Both Plk2 and Plk3 are proposed to function as tumor suppressors (Smith et al. 2006; Yang et al. 2008). On the other hand, Plk4 has been shown to function as a key regulator of centriole biogenesis at the early stage of the cell cycle (Bettencourt-Dias et al. 2005; Habedanck et al. 2005; Duensing et al. 2007; Kleylein-Sohn et al. 2007), suggesting that Plk4-dependent centriole duplication lays a groundwork for Plk1-dependent centrosome maturation and bipolar spindle formation at the time of mitotic entry.

### Plk1: a cancer cell-selective anticancer drug target

**Fig. 2:**
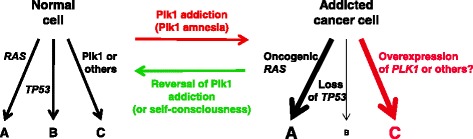
A schematic diagram illustrating hypothetical biochemical pathways between normal cells and Plk1-addicted cancer cells bearing an oncogenic *RAS* and/or a *TP53* loss-of-function mutation. Reversal of Plk1 addiction (or gaining self-consciousness from Plk1 amnesia) may induce cancer cell–selective mitotic arrest and apoptotic cell death

Consistent with the important role of Plk1 in regulating various mitotic events, Plk1 overexpression is thought to promote neoplastic transformation of human cells (Eckerdt et al. 2005; Strebhardt and Ullrich 2006; Strebhardt 2010). Not surprisingly, Plk1 overexpression appears to be tightly associated with aggressiveness and poor prognosis of various types of human cancers. In addition, recent genome-wide studies have revealed that Plk1 and a number of other mitotically important regulators, such as the anaphase-promoting complex/cyclosomes and the proteasome, are required for the viability of activated *RAS* or inactivated *TP53* mutation-bearing cancer cells, but not for the respective normal cells (Luo et al. 2009a; Sur et al. 2009). These observations suggest that cancer cells are addicted not only to oncogenic *RAS* or the inactivated p53 function, as Bernard Weinstein originally proposed (Weinstein 2002), but also to non-oncogenic Plk1, whose inhibition results in prometaphase accumulation and subsequent death (Luo et al. 2009b) (Fig. 2). These observations suggest that Plk1-dependent biochemical steps and signaling pathways are likely reprogrammed for the survival and proliferation of Plk1-addicted cancer cells. Under these conditions, the reversal of Plk1 addiction may be sufficient for triggering cancer cell-selective mitotic block and apoptotic cell death (Luo et al. 2009b), as has been demonstrated by the reversal of oncogene addictions (McMurray et al. 2008). As an alternative explanation to the oncogene (and also perhaps non-oncogene) addiction, Dean Felsher proposed that oncogene activation may induce a state of cellular amnesia, which allows cells to bypass surveillance mechanisms and, therefore, permits unregulated cell proliferation (Felsher 2008). Whether the altered cellular homeostasis in cancer cells is called addiction or amnesia, studies suggest that the reversal of Plk1 addiction is sufficient for inducing selective cellular senescence or apoptosis in oncogenic *RAS*- or inactivated *TP53*-containing cancer cells (Luo et al. 2009a; Sur et al. 2009) (Fig. 2). Therefore, antagonizing the Plk1 function appears to be a particularly appealing strategy for killing oncogenic *RAS-* or inactivated *TP53*-containing cancer cells. Since both Plk2 and Plk3 are required for promoting cell survival (Burns et al. 2003; Xie et al. 2005) and they exhibit properties similar to tumor suppressors (Smith et al. 2006; Yang et al. 2008; Coley et al. 2012), specific inhibition of Plk1, but not Plk2 or Plk3, would be important for selectively killing cancer cells, but not normal cells.

### Targeting the kinase domain vs. the PBD of Plk1

**Fig. 3:**
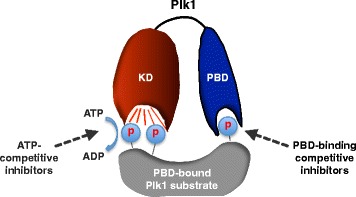
A schematic diagram illustrating two distinct strategies for targeting Plk1. ATP-competitive analogs have been widely developed to inhibit the catalytic activity of Plk1, while PBD-binding antagonists are being developed to competitively inhibit the function of PBD. *KD* kinase domain, *PBD* polo-box domain. In the diagram, the PBD is shown to interact with a phosphorylated epitope on a substrate, and it tethers the cis-acting N-terminal KD in close proximity to its substrate. *ATP* adenosine triphosphate, *ADP* adenosine diphosphate

Both the kinase domain (KD) and PBD of Plk1 are essentially required for the mitotic functions of Plk1 (Lee et al. 1998; Seong et al. 2002; Hansen et al. 2004), suggesting that they represent two distinct drug targets within one molecule (Fig. 3). The ATP-binding pocket within the KD serves as a well-defined target site that may allow one to achieve the complete annihilation of the kinase’s catalytic activity. Consequently, targeting the ATP-binding site has long been a prevailing method for generating kinase inhibitors. Over the years, several ATP analog inhibitors (e.g., BI2536, BI6727, GSK461364A, cyclapolin 1, DAP81, and TAL) have been developed to competitively inhibit Plk1 catalytic activity. BI6727 (also called volasertib) is the most advanced and has been evaluated under phase III clinical trials. However, because of the structural similarities among the catalytic domains of >500 intracellular kinases, these inhibitors largely exhibit a broad inhibitory activity against closely related Plk2 and Plk3 and several other kinases (Steegmaier et al. 2007; Rudolph et al. 2009; Raab et al. 2014), therefore frequently limiting their in vivo applicability because of poor pharmacological safety profiles and dose-limiting toxicity (Lee et al. 2015).

As a new approach to bypass the problems associated with currently available KD inhibitors, a large body of studies has been performed to generate Plk1 PBD antagonists. This new approach is important because targeting protein–protein interactions is considered a highly attractive strategy that provides a potential for developing specific inhibitors against a particular protein. However, finding a targetable protein–protein interaction motif with a distinct binding nature is thought to be difficult because binding surfaces are mostly nondescript. Against the odds, studies with Plk1 PBD revealed that a small and specific phosphopeptide is sufficient for interacting with Plk1 PBD, but not with PBDs from Plk2 or Plk3, with a high affinity (Elia et al. 2003; Yun et al. 2009), suggesting that specific inhibition of Plk1 PBD could be achieved by low-molecular weight, peptide-derived inhibitors, or structurally related compounds. Moreover, recent data suggests that interrogating Plk1 PBD function by expressing a highly specific, suicidal PBD-binding peptide can potently induce mitotic block and apoptosis in tumorigenic or metastatic cancer cells, but not in normal cells (Park et al. 2015). Albeit this promising outlook, small-molecule PBD inhibitors developed to date (Reindl et al. 2008; Watanabe et al. 2009) exhibit only suboptimal Plk1 PBD-binding affinity with an undefined binding mode (Liao et al. 2010). On the other hand, peptide-derived Plk1 PBD inhibitors exhibit superb binding affinity and specificity in vitro. However, they suffer greatly from poor membrane permeability and low bioavailability in cell-based assays (Liu et al. 2011; Qian et al. 2014), thus necessitating further improvement of these inhibitors for in vivo studies.

### Advantages of targeting the PBD of Plk1

Besides being an alternative target for anti-Plk1 drug discovery, it is important to note that inhibiting the PBD is fundamentally different from inhibiting the KD, at both physiological and biochemical levels. Studies showed that inhibiting Plk1 catalytic activity potently induces early mitotic arrest (Sumara et al. 2004; Hanisch et al. 2006; Lenart et al. 2007), whereas inhibiting Plk1 PBD function results in preanaphase arrest (Seong et al. 2002; Hanisch et al. 2006). These findings suggest that this Plk1 kinase activity-dependent process is essentially required from the early stage of mitosis, whereas the PBD-dependent Plk1 function is essential only at a much later stage but prior to the metaphase/anaphase transition. At the biochemical level, the KD binds to ATP as the only ligand, whereas the PBD binds to a large spectrum of binding targets, such as Plk1-binding proteins and physiological substrates (Park et al. 2010). Moreover, the PBD binds to its various targets with different levels of affinity, thus enabling the PBD to mediate diverse Plk1-dependent events in a differentially regulated manner. These fundamental differences in the biochemical properties of the KD versus the PBD predict that ATP analog inhibitors would annihilate Plk1 catalytic activity in both normal and cancer cells equally, whereas PBD inhibitors could interfere with only a subset of PBD-dependent interactions. Since Plk1-dependent signaling pathways and biochemical steps are predictably rewired in Plk1-addicted cancers, it would be feasible to tailor PBD inhibitors in such a way that they would interfere with PBD-dependent interactions enriched in cancer cells, but not normal cells. Therefore, unlike inhibiting Plk1 KD, antagonizing the PBD function may allow one to uniquely impose an additional layer of selectivity in killing cancer cells, but not normal cells.

### Recent advances in developing Plk1 PBD inhibitors

During the past several years, numerous independent efforts have been made to generate both small-molecule- and peptide-based PBD inhibitors (Lee et al. 2015). Small-molecule inhibitors were isolated from in vitro screenings designed to isolate compounds capable of disrupting the Plk1 PBD-dependent interaction with its cognate peptide ligand. These small-molecule inhibitors include a thymoquinone derivative, poloxin (Reindl et al. 2008), a benzylidene-thiazolotriazenedione derivative, poloxipan (Reindl et al. 2009), and a benzotropolone derivative, purpurogallin (Watanabe et al. 2009). However, these inhibitors exhibit only weak inhibitory activity against Plk1 PBD in vitro (Liao et al. 2010), with a substantial level of nonspecific toxicity in cultured cells (Park, J. -E, and K. S. Lee, unpublished data), suggesting that their applicability is likely limited. Optimizing these inhibitors to improve both Plk1 PBD-binding affinity and specificity would be necessary in order to use them in preclinical and clinical studies. The co-crystal structures of Plk1 PBD, in complex with the structurally similar thymoquinone or the oxime fragment of poloxin (called poloxime), have been determined (Yin et al. 2013). The binding mode of thymoquinone or the oxime fragment may potentially serve as a template for anti-PBD drug discovery.

**Fig. 4:**
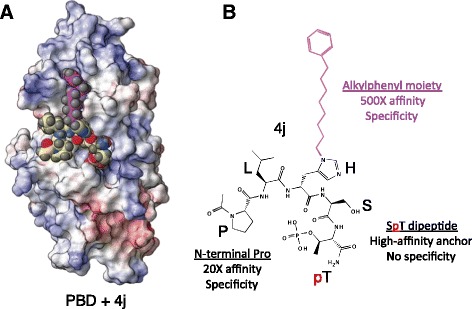
The mode of binding between Plk1 PBD and its high-affinity ligand, 4j. **a** Electrostatic surface representation of the Plk1 PBD in complex with 4j. Surface colors represent electrostatic potentials with *red* (*negative*), *blue* (*positive*), and *white* (*neutral*). For 4j, oxygen and nitrogen atoms are colored in *red* and *blue*, respectively. The alkylphenyl moiety is indicated in *violet*. The phosphorylated (*red*)-T residue is shown. **b** A schematic diagram showing 4j and its three elements, which are critical for Plk1 PBD binding. PLHSpT is shown in *single letters* except for phosphorylated (*red*)-T. The alkylphenyl moiety of 4j is indicated in *violet*

Studies with various PBD-binding proteins led to the identification of a peptide as small as 5-mer PLHSpT (Yun et al. 2009). This peptide was identified from the T78 motif of a kinetochore component called PBIP1 (Kang et al. 2006). PLHSpT falls into the consensus Plk1 PBD-binding target that was previously reported (Elia et al. 2003) and binds to Plk1 PBD with high affinity and specificity (*K*_d_ = 450 nM) (Yun et al. 2009). Over the years, substantial progress has been made in developing various PLHSpT derivatives with improved affinity and/or cellular activity (Liu et al. 2011, 2012a, b; Qian et al. 2012, 2014; Murugan et al. 2013a, b; Srinivasrao et al. 2014; Ahn et al. 2015). Among these derivatives, a C_6_H_5_(CH_2_)_8_ group-conjugated *4j* (Fig. 4a) is considered a prototype, and it exhibits dramatically increased (~500-fold) affinity (*K*_d_ = 1–2 nM) with an undiminished level of Plk1-binding specificity (Liu et al. 2011). The unexpected binding mode between the alkylphenyl moiety of *4j* and neighboring hydrophobic residues of Plk1 PBD may provide a new paradigm in the development of Plk1 PBD-binding inhibitors. In addition, the N-terminal Pro motif has been shown to confer Plk1 specificity and ~20-fold affinity to Plk1 PBD, while the C-terminal SpT dipeptide functions as a high-affinity anchor that is crucially required for establishing a stable interaction with the H538 and K540 residues of Plk1 PBD (Yun et al. 2009) (Fig. 4b). Although they exhibit superb binding affinity and specificity, *4j* and its derived inhibitors (Liu et al. 2011, 2012a, b; Qian et al. 2012, 2014) still require high extracellular concentrations (IC_50_ = 80–320 μM) to delocalize Plk1 and induce mitotic block in cultured HeLa cells. This requirement appears to be largely due to these inhibitors’ less than acceptable level of membrane permeability and intracellular stability (Liu et al. 2011). Further derivatization and optimization of these inhibitors would be necessary to improve the bioavailability and efficacy of the compounds.

Whether improving peptide-based inhibitors can lead to the development of more clinically applicable PBD antagonists remain to be seen. Nevertheless, the novel binding interactions within the C_6_H_5_(CH_2_)_8_ group may likely be critical for the future design of PBD-binding antagonists. Based on the earlier success in generating a PLHSpT-based cyclic peptomer (Murugan et al. 2013b), developing *4j*-based cyclic peptides and their derivatives could be important not only to their direct use for in vitro and/or in vivo applications, but also to the design and development of more druggable small-molecule-based inhibitors.

### Conclusions

It is now well appreciated that targeting PBD constitutes a fascinating strategy that may lead to the development of a new class of Plk1 inhibitors. Although substantial progress has been made in developing Plk1 PBD inhibitors during the last several years, these inhibitors suffer greatly from low binding affinity, poor specificity, or low membrane permeability. Nevertheless, studies with peptide-based inhibitors revealed that high-affinity and high-specificity binding to Plk1 PBD can be achieved by several structural motifs, such as the SpT high-affinity anchor, the N-terminal Pro moiety, and the recently discovered hydrophobic channel-based interactions. At present, one of the biggest challenges in developing *4j*-based inhibitors is improving membrane permeability by minimizing the anionic change of the phosphorylated Thr residue that is critical for PBD binding. Although developing *4j*-based inhibitors may have its own merits, it may be important to design and develop next-generation small-molecule PBD inhibitors that mimic the structure of *4j* or its derivatives.

## References

[CR1] Ahn M, Han YH, Park JE, Kim S, Lee WC, Lee SJ, Gunasekaran P, Cheong C, Shin SY, Kim HY, Ryu EK, Murugan RN, Kim NH, Bang JK (2015). A new class of peptidomimetics targeting the polo-box domain of polo-like kinase 1. J Med Chem.

[CR2] Archambault V, Glover DM (2009). Polo-like kinases: conservation and divergence in their functions and regulation. Nat Rev Mol Cell Biol.

[CR3] Arnaud L, Pines J, Nigg EA (1998). GFP tagging reveals human polo-like kinase 1 at the kinetochore/centromere region of mitotic chromosomes. Chromosoma.

[CR4] Bahassi EM, Conn CW, Myer DL, Hennigan RF, McGowan CH, Sanchez Y, Stambrook PJ (2002). Mammalian polo-like kinase 3 (Plk3) is a multifunctional protein involved in stress response pathways. Oncogene.

[CR5] Bettencourt-Dias M, Rodrigues-Martins A, Carpenter L, Riparbelli M, Lehmann L, Gatt MK, Carmo N, Balloux F, Callaini G, Glover DM (2005). SAK/PLK4 is required for centriole duplication and flagella development. Curr Biol.

[CR6] Burns TF, Fei P, Scata KA, Dicker DT, El-Deiry WS (2003). Silencing of the novel p53 target gene Snk/Plk2 leads to mitotic catastrophe in paclitaxel (taxol)-exposed cells. Mol Cell Biol.

[CR7] Chase D, Feng Y, Hanshew B, Winkles JA, Longo DL, Ferris DK (1998). Expression and phosphorylation of fibroblast-growth-factor-inducible kinase (Fnk) during cell-cycle progression. Biochem J.

[CR8] Cheng KY, Lowe ED, Sinclair J, Nigg EA, Johnson LN (2003). The crystal structure of the human polo-like kinase-1 polo box domain and its phospho-peptide complex. EMBO J.

[CR9] Coley HM, Hatzimichael E, Blagden S, McNeish I, Thompson A, Crook T, Syed N (2012). Polo like kinase 2 tumour suppressor and cancer biomarker: new perspectives on drug sensitivity/resistance in cancer. Oncotarget.

[CR10] Donohue PJ, Alberts GF, Guo Y, Winkles JA (1995). Identification by targeted differential display of an immediate early gene encoding a putative serine/threonine kinase. J Biol Chem.

[CR11] Duensing A, Liu Y, Perdreau SA, Kleylein-Sohn J, Nigg EA, Duensing S (2007). Centriole overduplication through the concurrent formation of multiple daughter centrioles at single maternal templates. Oncogene.

[CR12] Eckerdt F, Yuan J, Strebhardt K (2005). Polo-like kinases and oncogenesis. Oncogene.

[CR13] Elia AE, Rellos P, Haire LF, Chao JW, Ivins FJ, Hoepker K, Mohammad D, Cantley LC, Smerdon SJ, Yaffe MB (2003). The molecular basis for phospho-dependent substrate targeting and regulation of Plks by the polo-box domain. Cell.

[CR14] Felsher DW (2008). Oncogene addiction versus oncogene amnesia: perhaps more than just a bad habit?. Cancer Res.

[CR15] Golsteyn RM, Mundt KE, Fry AM, Nigg EA (1995). Cell cycle regulation of the activity and subcellular localization of Plk1, a human protein kinase implicated in mitotic spindle function. J Cell Biol.

[CR16] Habedanck R, Stierhof YD, Wilkinson CJ, Nigg EA (2005). The polo kinase Plk4 functions in centriole duplication. Nat Cell Biol.

[CR17] Hanisch A, Wehner A, Nigg EA, Sillje HH (2006). Different Plk1 functions show distinct dependencies on polo-box domain-mediated targeting. Mol Biol Cell.

[CR18] Hanks SK, Quinn AM, Hunter T (1988). The protein kinase family: conserved features and deduced phylogeny of the catalytic domains. Science.

[CR19] Hansen DV, Loktev AV, Ban KH, Jackson PK (2004). Plk1 regulates activation of the anaphase promoting complex by phosphorylating and triggering SCFbetaTrCP-dependent destruction of the APC inhibitor Emi1. Mol Biol Cell.

[CR20] Holtrich U, Wolf G, Bräuninger A, Karn T, Böhme B, Rübsamen-waigmann H, Strebhardt K (1994). Induction and down-regulation of PLK, a human serine/threonine kinase expressed in proliferating cells and tumors. Proc Natl Acad Sci U S A.

[CR21] Kang YH, Park J-E, Yu L-R, Soung N-K, Yun S-M, Bang JK, Seong YS, Yu H, Veenstra TD, Lee KS (2006). Self-regulation of Plk1 recruitment to the kinetochores is critical for chromosome congression and spindle checkpoint signaling. Mol Cell.

[CR22] Kleylein-Sohn J, Westendorf J, Le Clech M, Habedanck R, Stierhof YD, Nigg EA (2007). Plk4-induced centriole biogenesis in human cells. Dev Cell.

[CR23] Lahiry P, Torkamani A, Schork NJ, Hegele RA (2010). Kinase mutations in human disease: interpreting genotype–phenotype relationships. Nat Rev Genet.

[CR24] Lee KS, Yuan Y-L, Kuriyama R, Erikson RL (1995). Plk is an M-phase-specific protein kinase and interacts with a kinesin-like protein, CHO1/MKLP-1. Mol Cell Biol.

[CR25] Lee KS, Grenfell TZ, Yarm FR, Erikson RL (1998). Mutation of the polo-box disrupts localization and mitotic functions of the mammalian polo kinase Plk. Proc Natl Acad Sci U S A.

[CR26] Lee KS, Song S, Erikson RL (1999). The polo-box-dependent induction of ectopic septal structures by a mammalian polo kinase, Plk, in *Saccharomyces cerevisiae*. Proc Natl Acad Sci U S A.

[CR27] Lee KS, Burke TRJ, Park J-E, Bang JK, Lee E. Recent advances and new strategies in targeting Plk1 for anticancer therapy. Trends Pharmacol Sci. 2015. (in press). 10.1016/j.tips.2015.08.013 PMC4684765 26478211

[CR28] Lenart P, Petronczki M, Steegmaier M, Di Fiore B, Lipp JJ, Hoffmann M, Rettig WJ, Kraut N, Peters JM (2007). The small-molecule inhibitor BI 2536 reveals novel insights into mitotic roles of polo-like kinase 1. Curr Biol.

[CR29] Leung GC, Hudson JW, Kozarova A, Davidson A, Dennis JW, Sicheri F (2002). The Sak polo-box comprises a structural domain sufficient for mitotic subcellular localization. Nat Struct Biol.

[CR30] Liao C, Park JE, Bang JK, Nicklaus MC, Lee KS (2010). Exploring potential binding modes of small drug-like molecules to the polo-box domain of human polo-like kinase 1. ACS Med Chem Lett.

[CR31] Liu F, Park JE, Qian WJ, Lim D, Graber M, Berg T, Yaffe MB, Lee KS, Burke TR (2011). Serendipitous alkylation of a Plk1 ligand uncovers a new binding channel. Nat Chem Biol.

[CR32] Liu F, Park JE, Qian WJ, Lim D, Scharow A, Berg T, Yaffe MB, Lee KS, Burke TRJ (2012). Identification of high affinity polo-like kinase 1 (Plk1) polo-box domain binding peptides using oxime-based diversification. ACS Chem Biol.

[CR33] Liu F, Park JE, Qian WJ, Lim D, Scharow A, Berg T, Yaffe MB, Lee KS, Burke TRJ (2012). Peptoid-peptide hybrid ligands targeting the polo box domain of polo-like kinase 1. Chembiochem.

[CR34] Lowery DM, Mohammad DH, Elia AE, Yaffe MB (2004). The polo-box domain: a molecular integrator of mitotic kinase cascades and polo-like kinase function. Cell Cycle.

[CR35] Luo J, Emanuele MJ, Li D, Creighton CJ, Schlabach MR, Westbrook TF, Wong KK, Elledge SJ (2009). A genome-wide RNAi screen identifies multiple synthetic lethal interactions with the Ras oncogene. Cell.

[CR36] Luo J, Solimini NL, Elledge SJ (2009). Principles of cancer therapy: oncogene and non-oncogene addiction. Cell.

[CR37] Ma S, Charron J, Erikson RL (2003). Role of Plk2 (Snk) in mouse development and cell proliferation. Mol Cell Biol.

[CR38] Ma S, Liu MA, Yuan Y-L, Erikson RL (2003). The serum-inducible protein kinase Snk is a G1 phase polo-like kinase that is inhibited by the calcium- and integrin-binding protein CIB. Mol Cancer Res.

[CR39] McMurray HR, Sampson ER, Compitello G, Kinsey C, Newman L, Smith B, Chen SR, Klebanov L, Salzman P, Yakovlev A, Land H (2008). Synergistic response to oncogenic mutations defines gene class critical to cancer phenotype. Nature.

[CR40] Murugan RN, Ahn M, Lee WC, Kim HY, Song JH, Cheong C, Hwang E, Seo JH, Shin SY, Choi SH, Park JE, Bang JK (2013). Exploring the binding nature of pyrrolidine pocket-dependent interactions in the polo-box domain of polo-like kinase 1. PLoS One.

[CR41] Murugan RN, Park JE, Lim D, Ahn M, Cheong C, Kwon T, Nam KY, Choi SH, Kim BY, Yoon DY, Yaffe MB, Yu DY, Lee KS, Bang JK (2013). Development of cyclic peptomer inhibitors targeting the polo-box domain of polo-like kinase 1. Bioorg Med Chem.

[CR42] Park JE, Soung NK, Johmura Y, Kang YH, Liao C, Lee KH, Park CH, Nicklaus MC, Lee KS (2010). Polo-box domain: a versatile mediator of polo-like kinase function. Cell Mol Life Sci.

[CR43] Park S-Y, Park J-E, Kim T-S, Kim JH, Kwak M-J, Ku B, Tian L, Murugan RN, Ahn M, Komiya S, Hojo H, Kim NH, Kim BY, Bang JK, Erikson RL, Lee KY, Kim SJ, Oh B-H, Yang W, Lee KS (2014). Molecular basis for unidirectional scaffold switching of human Plk4 in centriole biogenesis. Nat Struct Mol Biol.

[CR44] Park J-E, Kim TS, Lee KS. Selective blockade of cancer cell proliferation and anchorage-independent growth by Plk1 activity–dependent suicidal inhibition of its polo-box domain. Cell Cycle. 2015. (in press). 10.1080/15384101.2015.1104435 PMC4825759 26513691

[CR45] Petronczki M, Lénárt P, Peters JM (2008). Polo on the rise-from mitotic entry to cytokinesis with Plk1. Dev Cell.

[CR46] Qian WJ, Park JE, Lee KS, Burke TR (2012). Non-proteinogenic amino acids in the pThr-2 position of a pentamer peptide that confer high binding affinity for the polo box domain (PBD) of polo-like kinase 1 (Plk1). Bioorg Med Chem Lett.

[CR47] Qian WJ, Park JE, Lim D, Lai CC, Kelley JA, Park SY, Lee KW, Yaffe MB, Lee KS, Burke TR (2014). Mono-anionic phosphopeptides produced by unexpected histidine alkylation exhibit high plk1 polo-box domain-binding affinities and enhanced antiproliferative effects in hela cells. Biopolymers.

[CR48] Raab M, Pachl F, Kramer A, Kurunci-Csacsko E, Dotsch C, Knecht R, Becker S, Kuster B, Strebhardt K (2014). Quantitative chemical proteomics reveals a Plk1 inhibitor-compromised cell death pathway in human cells. Cell Res.

[CR49] Reindl W, Yuan J, Krämer A, Strebhardt K, Berg T (2008). Inhibition of polo-like kinase 1 by blocking polo-box domain-dependent protein-protein interactions. Chem Biol.

[CR50] Reindl W, Yuan J, Kramer A, Strebhardt K, Berg T (2009). A pan-specific inhibitor of the polo-box domains of polo-like kinases arrests cancer cells in mitosis. Chembiochem.

[CR51] Rudolph D, Steegmaier M, Hoffmann M, Grauert M, Baum A, Quant J, Haslinger C, Garin-Chesa P, Adolf GR (2009). BI 6727, a polo-like kinase inhibitor with improved pharmacokinetic profile and broad antitumor activity. Clin Cancer Res.

[CR52] Seong YS, Kamijo K, Lee JS, Fernandez E, Kuriyama R, Miki T, Lee KS (2002). A spindle checkpoint arrest and a cytokinesis failure by the dominant-negative polo-box domain of Plk1 in U-2 OS cells. J Biol Chem.

[CR53] Simmons DL, Neel BG, Stevens R, Evett G, Erikson RL (1992). Identification of an early-growth-response gene encoding a novel putative protein kinase. Mol Cell Biol.

[CR54] Smith P, Syed N, Crook T (2006). Epigenetic inactivation implies a tumor suppressor function in hematologic malignancies for polo-like kinase 2 but not polo-like kinase 3. Cell Cycle.

[CR55] Srinivasrao G, Park JE, Kim S, Ahn M, Cheong C, Nam KY, Gunasekaran P, Hwang E, Kim NH, Shin SY, Lee KS, Ryu E, Bang JK (2014). Design and synthesis of a cell-permeable, drug-like small molecule inhibitor targeting the polo-box domain of polo-like kinase 1. PLoS One.

[CR56] Steegmaier M, Hoffmann M, Baum A, Lénárt P, Petronczki M, Krssák M, Gürtler U, Garin-Chesa P, Lieb S, Quant J, Grauert M, Adolf GR, Kraut N, Peters JM, Rettig WJ (2007). BI 2536, a potent and selective inhibitor of polo-like kinase 1, inhibits tumor growth in vivo. Curr Biol.

[CR57] Strebhardt K (2010). Multifaceted polo-like kinases: drug targets and antitargets for cancer therapy. Nat Rev Drug Discov.

[CR58] Strebhardt K, Ullrich A (2006). Targeting polo-like kinase 1 for cancer therapy. Nat Rev Cancer.

[CR59] Sumara I, Gimenez-Abian JF, Gerlich D, Hirota T, Kraft C, de la Torre C, Ellenberg J, Peters JM (2004). Roles of polo-like kinase 1 in the assembly of functional mitotic spindles. Curr Biol.

[CR60] Sur S, Pagliarini R, Bunz F, Rago C, Diaz LAJ, Kinzler KW, Vogelstein B, Papadopoulos N (2009). A panel of isogenic human cancer cells suggests a therapeutic approach for cancers with inactivated p53. Proc Natl Acad Sci U S A.

[CR61] Watanabe N, Sekine T, Takagi M, Iwasaki J, Imamoto N, Kawasaki H, Osada H (2009). Deficiency in chromosome congression by the inhibition of Plk1 polo box domain-dependent recognition. J Biol Chem.

[CR62] Weinstein IB (2002). Cancer. Addiction to oncogenes—the Achilles heal of cancer. Science.

[CR63] Winkles JA, Alberts GF (2005). Differential regulation of polo-like kinase 1, 2, 3, and 4 gene expression in mammalian cells and tissues. Oncogene.

[CR64] Xie S, Wang Q, Wu H, Cogswell J, Lu L, Jhanwar-Uniyal M, Dai W (2001). Reactive oxygen species-induced phosphorylation of p53 on serine 20 is mediated in part by polo-like kinase-3. J Biol Chem.

[CR65] Xie S, Wu H, Wang Q, Cogswell JP, Husain I, Conn C, Stambrook P, Jhanwar-Uniyal M, Dai W (2001). Plk3 functionally links DNA damage to cell cycle arrest and apoptosis at least in part via the p53 pathway. J Biol Chem.

[CR66] Xie S, Wu H, Wang Q, Kunicki J, Thomas RO, Hollingsworth RE, Cogswell J, Dai W (2002). Genotoxic stress-induced activation of Plk3 is partly mediated by Chk2. Cell Cycle.

[CR67] Xie S, Xie B, Lee MY, Dai W (2005). Regulation of cell cycle checkpoints by polo-like kinases. Oncogene.

[CR68] Yang Y, Bai J, Shen R, Brown SA, Komissarova E, Huang Y, Jiang N, Alberts GF, Costa M, Lu L, Winkles JA, Dai W (2008). Polo-like kinase 3 functions as a tumor suppressor and is a negative regulator of hypoxia-inducible factor-1 alpha under hypoxic conditions. Cancer Res.

[CR69] Yin Z, Song Y, Rehse PH (2013). Thymoquinone blocks pSer/pThr recognition by Plk1 polo-box domain as a phosphate mimic. ACS Chem Biol.

[CR70] Yun SM, Moulaei T, Lim D, Bang JK, Park JE, Shenoy SR, Liu F, Kang YH, Liao C, Soung NK, Lee S, Yoon DY, Lim Y, Lee DH, Otaka A, Appella E, McMahon JB, Nicklaus MC, Burke TR, Yaffe MB, Wlodawer A, Lee KS (2009). Structural and functional analyses of minimal phosphopeptides targeting the polo-box domain of polo-like kinase 1. Nat Struct Mol Biol.

[CR71] Zitouni S, Nabais C, Jana SC, Guerrero A, Bettencourt-Dias M (2014). Polo-like kinases: structural variations lead to multiple functions. Nat Rev Mol Cell Biol.

